# Dietary diversity determinants and contribution of fish to maternal and under-five nutritional status in Zambia

**DOI:** 10.1371/journal.pone.0204009

**Published:** 2018-09-24

**Authors:** Pamela A. Marinda, Sven Genschick, Christopher Khayeka-Wandabwa, Rebecca Kiwanuka-Lubinda, Shakuntala H. Thilsted

**Affiliations:** 1 The University of Zambia, School of Agricultural Sciences, Department of Food Science and Nutrition, Lusaka, Zambia; 2 WorldFish, Lusaka, Zambia; 3 School of Pharmaceutical Science and Technology (SPST), Health Science Platform, Tianjin University, Tianjin city, Nankai District, China; 4 The University of Zambia, School of Agricultural Sciences, Department of Agricultural Economics and Extension, Lusaka, Zambia; 5 WorldFish, Penang, Malaysia; Institut de recherche pour le developpement, FRANCE

## Abstract

**Background:**

This study examines socio-economic determinants of food consumption patterns amongst women of reproductive age and children aged 6–59 months from urban poor settlements of Lusaka and their implications for nutritional status. Particular emphasis was placed on the role of fish in their diets and nutritional status.

**Methods:**

A cross-sectional survey design was applied, in which 714 mother-child dyads, with children aged 6–59 months were enrolled. A three-stage randomized cluster sampling approach was applied.

**Results:**

The mean dietary diversity score among children aged 6–23 and 24–59 months was 2.98 (±1.27) and 3.478 (±1.07), respectively. In children aged 6–23 months, there was a significant difference in their nutritional status, based on fish consumption (χ^2^ = 10.979, df = 2, p = 0.004). Children from poorer households consumed mostly small fish (*Kapenta*). The quantity of fish consumed by children was significantly associated with stunting in both age groups, odds ratio = 0.947 (95% CI: 0.896, 1.000) for children aged 6–23 months and odds ratio = 1.038 (95% CI: 1.006, 1.072) for children aged 24–59 months old. Other significant risk factors for stunting in children aged 6–23 months were the child’s age, mother’s body mass index, access to treated water and child morbidity. Child’s age, mother’s educational level and wealth status were determinants of dietary diversity in children aged 6–59 months as shown by the Poisson regression.

**Conclusion:**

Nutritional status of children aged 6–23 months is associated with fish consumption, with children consuming fish less likely to be stunted. Small fish (*Kapenta*) is an animal-source food that is particularly important in the diet of children in urban poor households in Zambia and contributes to better nutritional outcomes. As all small fish stem from capture fisheries, sustainable one health environmental integration, monitoring and management strategies are desirable.

## Introduction

The Sustainable Development Goals (SDGs) 2 and 14 outline achieving food and nutrition security and ending malnutrition as global priorities by focusing on the significance of sustaining food production and securing year-round access to diverse foods [1]. In order to optimally harness from the two SDGs guiding pillars and anticipated benefits in the 21^st^ century global health dynamics, it is increasingly becoming imperative to track dietary diversity, quality and nutritional outcomes in the diverse global population segments [2]. These commitments bring to the forefront insights into nutritional status and dietary intake of populations as well as indicators for food consumption and diet quality. In addition, desired health interventions needs and progressive multi-sectorial policy guide essentials towards food and nutrition security are tenable through such efforts[2,3]. The World Health Organization (WHO) estimates that undernutrition is directly or indirectly responsible for at least 35% of the deaths in children under the age of five years, globally[4]. Zambia, like many other developing countries, is currently faced with a double burden of malnutrition, with undernutrition continuing to be a major public health issue. According to the Zambia Demographic Health Survey (DHS) 2013–2014, prevalence of stunting in children under five years is 40%, underweight 15% and wasting 5% [5] and acute and chronic micronutrient deficiencies, particularly vitamin A, iron, zinc and iodine deficiencies, exist in high proportions in both rural and urban areas [6].

Numerous studies indicate that inadequate food/nutrient intake is one of the major factors responsible for undernutrition. Inadequacies in complementary feeding are common, with foods of low nutrient density and with little consumption of animal-source foods in low-income households [7–10]. Animal-source foods are seldom consumed in adequate amounts, especially by the poor. Among animal-source foods, the importance of fish for food and nutrition security is increasingly being recognized, as is its emerging potential in addressing hidden hunger. The Food and Agriculture Organization (FAO) reports that fish and seafood now contribute to 15% of average animal protein consumed by three billion people worldwide, fisheries and aquaculture directly employ about 45 million people and an estimated five hundred and forty million people derive their livelihoods from seafood-related industries [11,12]. Furthermore, the expansion of fish supply through aquaculture has significantly contributed to higher penetration of fish/seafood across urban and rural areas, including regions where people have traditionally eaten little fish. In the Scaling Up Nutrition (SUN) Framework and Roadmap: 1,000 Days Global Effort, the importance of essential fatty acids for brain development and cognition, affecting individual, national and global development, as well as fish being a rich source of essential fats have been highlighted [13]. The Copenhagen Consensus 2012 Expert Panel ranked “bundled micronutrients interventions to fight hunger and improve nutrition” as number one of the 16 investments worthy of investment for global development [14–16]. Fish, in particular small fish, fresh or dried, are commonly consumed in small quantities by the poor. However, small fish is an overlooked rich source of multiple essential micronutrients such as vitamin B12, vitamin A, iron, zinc and calcium which are lacking in the diet [17,18].

Social factors have also been found to be associated with nutritional status in developing countries, for example, Kenya and Ghana [19,20], and the relationships between these social determinants and nutritional status have been found to change over time [21]. Food choices and food consumption patterns are influenced by a number of factors that include: gender, socio-economic factors, level of education, nutritional knowledge, and social and cultural factors [22–24]. For children, complementary food choices are made by their mothers or caregivers. Some mothers and caregivers have poor understanding of what constitutes a healthy diet and some studies have demonstrated that mothers' food choices for their children may be influenced by the child’s gender[25]. Demographic and socio-economic factors such as household wealth, maternal education, parity, mother's age, child's age, pregnancy history and time for preparing foods and feeding the child have been found to influence health seeking behaviors and optimal child care practices [26], thereby affecting child nutritional status.

The presented findings examine food consumption patterns amongst women and children aged 6–59 months in households from urban poor settlements of Lusaka, Zambia and their implications for nutritional status. Research questions addressed are: (1) whether socio-economic factors influence food consumptions patterns of women and under-five children from urban poor households; (2) to what extent fish and fish products contribute to diets in urban poor households; and (3) whether any associations exist between dietary intake, fish consumption and nutrition status of women and children aged 6–59 months.

## Materials and methods

### Study setting

Lusaka district in Lusaka Province was purposively selected as the study area for the following reasons: it is an urban area within Lusaka Province with the highest number of high density settlement townships where the majority of the urban poor live in Zambia. The study targeted low-income settlement localities as the people living in these areas are most vulnerable to food and nutrition insecurity. Three constituencies: Kanyama, Matero and Munali, from Lusaka district were purposively selected. According to the Zambia Living Condition Monitoring Survey [27,28], a high proportion of households from Kanyama (68%), Matero (65%) and Munali (35.6%) were classified as “near poor”, “poor” or “extremely poor”. The majority of people living in these areas do not engage in any agricultural activities and rely on the market for their food supplies.

### Study design and target population

Using a cross-sectional study design, a household survey was conducted to establish the food consumption patterns and nutritional status among women of reproductive age (WRA) (15–49 years old) and having children aged 6–59 months. Thus, households with mother-child dyads were targeted.

### Sample size determination and sampling procedure

To derive the sample size,

$$n=\frac{Z^{2}\alpha_{/2}\pi (1-\pi )}{d^{2}}$$

was applied; n is the minimum required sample size, Z is the Z score for the desired level of confidence (assumed to be 95% or *α* = 0.05), *π* is the population proportion of interest estimated to be 11%, the prevalence of stunted growth among children in Lusaka [27,28] and *d* is the margin of error (assumed to be 5%). The calculated sample size was further adjusted for the design effect and non-response rate (predicted to be 5%), to obtain the optimal sample size of 714 households. A sampling frame was developed from the 2010 Population Census and Housing report, in consultation with the local authorities and the Central Statistics Office (CSO). The sampling process involved, firstly, purposively selecting the three constituencies (Kanyama, Matero and Munali) from Lusaka district. From each constituency, one ward was randomly selected to participate in the study. In each reporting domain, study households were selected using a three-stage randomized cluster approach, with the first two stages using the Ward and Standard Enumeration Area (SEA) sampling frame from the 2010 CSO. A total of 36 SEAs (clusters) were identified and from each, 20 households were selected. Using a determined sampling interval, systematic random sampling was used in the final sampling stage. The first household that met the inclusion criteria (i.e. with mother-child dyad and with children aged 6–23 months and/or 24–59 months) was randomly picked. Using the sampling interval, the next households were picked and this procedure was followed until the required number of households was obtained. In households with more than one woman and/or child in each age group, one woman and one child in each age group were randomly selected.

Preceding data collection, enumerators with prior experience on household surveys were trained on how to conduct the survey, take anthropometric measures and collect dietary data. The data collection tools were pre-tested and amendments made.

### Data collection tool and procedures

A standard questionnaire specific to the scope of the study objectives was applied. The sub-sections included in the questionnaire are described below.

#### Demographic and socio-economic data

Data on the demographic characteristics of household members, income-earning activities, household assets, water and sanitary living conditions of the household were collected. Data on household assets were used to determine relative household wealth through a computed asset-based indicator, using Principal Component Analysis (PCA) [29].

#### Food consumption data

Food frequency questionnaire (FFQ) gathered dietary data on foods consumed and frequency of their consumption by women and children. The children’s FFQ had seven food groups, following guidelines from WHO and UNICEF [30]. The food groups are: 1) grains, roots or tubers 2) pulses, legumes or nuts, 3) flesh foods (meat, poultry and fish), 4) milk and milk products, 5) eggs, 6) vitamin A-rich fruit and vegetables, 7) Other fruit and vegetables. Food items consumed from the sugary food group (sweets and drinks) and fats and oils were also recorded but were excluded from the dietary diversity score (DDS) determination because they are poor in nutrients. The FFQ for women had ten (10) food groups, according to FAO and FHI360 criteria [31]; 1) grains, white roots and tubers, and plantains, 2) pulses (beans, peas and lentils), 3) nuts and seeds, 4) dairy, 5) meat, poultry and fish, 6. eggs, 7) dark green leafy vegetables, 8) other vitamin A-rich fruit and vegetables, 9) other vegetables, and 10) other fruit. Data obtained using the FFQ were used to determine the dietary diversity which is considered a proxy indicator for micronutrient adequacy of diets [30,31]. Additional questions on gender and age-specific fish consumption behaviour were included in the questionnaire, as well as questions on household members’ preferences for fish consumed (in terms of size, types, quality, and frequency of consumption of fish), fish in young children’s diet, that is, use of fish in the initiation of complementary feeding, the age at which children are first fed fish, amounts of fish consumed and the perceptions of the mothers’ on the importance of fish for growth and development of young children. A 24 hour recall was also used to collect data on amounts of specific food consumed in the preceding 24 hours. Of particular interest to this paper was data on amount of fish consumed by mothers and children aged 6–59 months.

#### Anthropometric measurements

Weight and length/height measurement were taken for all study children and women. Weight was taken using the SECA (S-876) electronic scale. Participants were weighed with minimal clothing to the nearest 0.1 kg. SECA length boards (Seca 417) and Stadiometer (Seca 217) were used to take the length and height to the nearest 0.1 cm, respectively. Measurements for each child and mother were taken twice and the average determined and recorded. Children’s age was verified using the under-five clinic card.

### Statistical analysis

Statistical Package for Social Sciences (SPSS, version 23) and STATA were used to analyze the data. WHO classification [32] was applied for the height-for-age, weight-for-height and weight-for-age Z scores cut-offs. Mothers’ body mass index (BMI) was based on WHO categorization [33]. For children, the 7 food groups were first summed into a score, ranging from 0 to 7. Each child was coded “yes = 1” for scoring ≥ 4 and “no = 0” for scoring less (0–3), for proportions of children who met or did not meet the minimum dietary diversity. The 10 food groups for women were first summed into a score, ranging from 0 to 10. Each woman was then coded “yes” for scoring ≥ 5 and “no” for scoring 0–4 for proportion of women who did or did not meet the minimum dietary diversity (MDD-W) as stipulated by FAO and FHI360 [30,31]. MDD-W is an indicator for whether women have consumed at least five out of 10 defined food groups during the previous day or night. At population level, the proportion of women 15–49 years of age who reach this minimum in a population can be used as a proxy indicator for high micronutrient adequacy in this age group. PCA was used to calculate the relative wealth index [34,35], the calculated standardized asset index score ranged between -2.4382 and 1.4016 and was used to compute quartiles (wealth groups). In addition, associations between various variables of interest were determined. Pearson Chi-Square test and t-test were used to establish statistically significant differences between selected variables. A logistic regression was used to establish determinants of child nutritional status and the contribution of fish consumption to children’s nutritional status. The model used was as follows: γ = α + β_*j*_*X*_*j*_ + *μ_j_* where, *y* is the independent variable (0 = child is stunted, and 1 = normal), *X_j_* is a vector of control and explanatory variables that include (i) characteristics of child j including age, gender of child, morbidity (health status) and infant feeding practices (breastfed or not; child’s dietary diversity), (ii) characteristics of child j’s mother including age, height, BMI, education (iii) household characteristics: household size, access to clean water and toilet and (iv) β_*j*_ are coefficients that were estimated. Stunting was selected as the dependent variable as it is a measure of long-term chronic undernutrition, a cumulative indicator of growth failure due to inadequate dietary intake, frequent infection, and sustained inappropriate feeding practices. Values of p < 0.05 were considered statistically significant.

### Ethics

The research protocol was approved by the University of Zambia Research Ethics Committee (UNZAREC). As data were collected electronically, a written informed consent was read out to potential study participants and signed by those who agreed to participate in the study. Participants who could not read and write gave a thumb-print on the consent form. Prior to starting the data collection exercise, the study objectives were explained to the respondents by the enumerators, who then proceeded to collect data, only from those who agreed to participate in the study.

## Results

### Demographic and socio-economic characteristics

The analyses were based on data from 714 households. Demographic and socio-economic characteristics are presented in Table 1. The poor are not a homogenous group, therefore, poor households were further disaggregated by material wealth, to study socio-economic factors and role of fish consumption in children’s diet, maternal and child nutritional status. Based on their relative wealth index, households were aggregated into four quartiles representing four socio-economic status groups (SES-G1-4): from poorest (SES-G1), first quartile, to the fourth quartile, relatively wealthy (SES-G4). Due to fewer unique asset index values than the overall size of the sample, numerous households showed almost the same asset index score which explains why the number of households among quartiles is not equally distributed. Between the quartiles, there was a gradual increase in the proportion of those who owned various household assets, from relatively low asset ownership in SES-G1 to relatively high asset ownership in SES-G4). Further wealth status insights are presented in findings that relied on the same household survey[36]. More than half of the mothers had primary level education; none had tertiary level education and the majority not having any form of formal employment. The mothers’ average age was 28.5 years (SE ±7.7) and most were married. A total of 759 children were enrolled in the study. Some households had children aged 6–23 months and aged 24–59 months. More than half of the children (52.7%, n = 399) were aged 24–59 months.

**Table 1:** Household demographic and socio-economic characteristics.

	n		%
**Household characteristics**			
**Wealth group (quartile)**			
1 (SES-G1)	175		24.5
2 (SES-G2)	175		24.5
3 (SES-G3)	171		23.9
4 (SES-G4)	193		27.0
**Characteristics of mothers**			
Mean age (years)	28.5		
**Educational level**			
No education	87		12.2
Primary	507		71.0
Secondary	120		16.8
Tertiary	0		0.0
**Marital status**			
Married	561		78.5
Single	102		14.3
Divorced/separated	27		3.8
Widowed	24		3.4
**Employment status**			
Informal	375		52.5
Formal	25		3.5
Unpaid household work (housewives)	314		44.0
**Characteristics of children**			
Age			
6–23 months	360		47.4
24–59 months	399		52.7
Average number of children <5 years	1.22		
Sex			
Female (aged 6–23 months)	210		58.3
Male (aged 6–23 months)	150		41.7
Female (aged 24–59 months)	210		52.6
Male (aged 24–59 months)	189		47.4

Socio-economic status group (SES-G1-4): Poorest (SES-G1), second (SES-G2), third (SES-G3), relatively wealthy (SES-G4).

### Food consumption by age and socio-economic status

Consumption of food items from various food groups in the preceding 24 hours was determined for children in both age groups (6–23 and 24–59 months). Foods from the starchy staples (cereals, grains, roots and tubers) were consumed by almost all children in both age categories. In children aged 6–23 months, the second most consumed foods were pulses, legumes and nuts (43.1%, n = 155), followed by flesh foods (meat, poultry, organ meat and fish) (33.1%, n = 119). Least consumed foods by children in this age group were eggs, vitamin A-rich fruit and vegetables (yellow and orange in colour), vitamin A-rich fruit and vegetables (dark green in colour) and other fruit and vegetables such as cucumber, eggplant and cabbage. A high proportion of children in the two age groups (6–23 and 24–59 months) consumed sugary foods (64.7 and 63.2%) and fatty foods (70.8% and 87.2%), respectively (Table 2). These foods are of low nutrient density. Consumption of sugary foods was usually in the form of biscuits and artificial juices, often sold along the streets in urban poor townships.

**Table 2:** Food groups consumed by children.

Food group	Children aged 6–23 months		Children aged 24–59 months	
	n	%	N	%
Starchy staples	352	97.8	397	99.5
Pulses, legumes and nuts	155	43.1	156	39.1
Milk and milk products	64	17.8	58	14.5
Flesh foods	119	33.1	207	51.9
Eggs	43	11.9	61	15.3
Vitamin A fruit and vegetables (yellow and orange in colour)	53	14.7	79	19.8
Vitamin A fruit and vegetables (dark green in colour)	97	26.9	208	52.1
Other fruit and vegetables	46	12.8	72	18
*Other groups (not considered in DDS calculation)*				
Oil, fat, butter	255	70.8	348	87.2
Sugary foods/sweets	233	64.7	262	63.2
Beverages	95	26.4	252	63.2

The DDS for children aged 6–23 months and those aged 24–59 months was determined (as described above). A child aged 6–23 months met the criterion for minimum dietary diversity if she/he had received food from four or more food groups [30]. The mean individual DDS for children aged 6–23 months was 2.98 (±1.27), and for children aged 24–59 months 3.48 (±1.07). Out of the 360 children aged 6–23 months, 35.6% (n = 128) met the minimum dietary diversity. For children aged 24–59 months, 48.6% (n = 194) met the minimum dietary diversity. Although this criterion was developed for children aged 6–23 months, in this study, it was also applied for children aged 24–59 months. Overall, more than half (57.6%, n = 437) of all children aged 6–59 months did not meet the acceptable minimum dietary diversity; 42.4% (n = 322) met the minimum dietary diversity.

Evaluating dietary diversity of children by wealth group, the proportions of children not meeting the minimum dietary diversity in SES-G1 and SES-G3 were higher compared to children in SES-G2 and SES-G4, for both age groups (Table 3). There was no statistically significant difference in dietary diversity by wealth group, for children aged 6–23 months (χ^2^ = 5.440, p = 0.142, df = 3). Chi square statistics showed a significant difference in dietary diversity by wealth groups among children aged 24–59 months (χ^2^ = 10.422, p = 0.015, df = 3).

**Table 3:** Proportion of children aged 6–59 months meeting/not meeting minimum dietary diversity per wealth group.

Wealth groups/ Age group	Meeting minimum dietary diversity		Not meeting minimum dietary diversity		Total	
Children 6–23 months (N = 360)	n	%	n	%	n	%
SES-G1	25	6.9	61	16.9	86	23.9
SES-G2	35	9.7	54	15.0	89	24.7
SES-G3	28	7.8	64	17.8	92	25.6
SES-G4	40	11.1	53	14.7	93	25.8
Total	128	35.6	232	64.4	360	100
Children 24–59 months (N = 399)						
SES-G1	39	9.8	60	15.0	99	24.8
SES-G2	44	11.0	49	12.3	93	23.3
SES-G3	43	10.8	52	13.0	95	23.8
SES-G4	68	17.0	44	11.0	112	28.1
Total	194	48.6	205	51.4	399	100

Socio-economic status group (SES-G1-4): Poorest (SES-G1), second (SES-G2), third (SES-G3), relatively wealthy (SES-G4).

#### Food consumption of food groups by women

Starchy staples were the most consumed food group by almost all women (99.4%). More than half of the women (59.9%) reported to have consumed flesh foods (poultry, meat, fish) in the preceding 24 hours, with a similar proportion (59.9%) having consumed dark green vegetables, whereas, only a small proportion of mothers had consumed dairy products, eggs, and nuts (Table 4). The majority of women also consumed low nutrient density foods: oils, fat and butter as well as sugary foods and beverages.

**Table 4:** Food consumption of food groups by women.

Food group	Consumed		Not consumed	
	N	%	N	%
Starchy staples	710	99.4	4	0.6
Pulses	215	30.1	499	69.9
Nuts and seeds	16	2.2	658	97.8
Milk and milk products	109	15.3	698	84.7
Flesh foods	428	59.9	286	40.1
Eggs	100	14.0	614	86.0
Vitamin A fruit and vegetables (yellow and orange in colour)	117	16.4	597	83.6
Dark green vegetables	427	59.8	287	40.2
Other fruit	141	19.7	573	80.3
Other vegetables	466	65.3	248	34.7

#### Dietary diversity in women

Of the 714 women who participated in the study, 87.5%, (n = 625) achieved minimum dietary diversity. The mean MDD-W was 5.8, with a range of 2.0 to 10.0. The proportion of women meeting the MDD-W was more likely to achieve adequate micronutrient intakes. Dietary diversity by wealth groups revealed no major differences in the proportions of women consuming food items from 5 or more food groups (χ^2^ = 7.69; df = 3; p = 0.053). About one quarter of women from each wealth group consumed food items from 5 or more food groups (Table 5).

**Table 5:** Minimum dietary diversity of women by wealth group.

Wealth group	Less than 5 food groups		5 or more food groups		Total		Pearson Chi-square *p-value*
	n	%	n	%	N	%	
SES-G1	31	4.3	144	20.2	175	24.5	0.053
SES-G2	23	3.2	148	20.7	171	23.9	
SES-G3	18	2.5	157	22.0	175	24.5	
SES-G4	17	2.4	176	24.7	193	25.1	
Total	89	12.5	625	87.5	714	100	

Socio-economic status group (SES-G1-4): Poorest (SES-G1), second (SES-G2), third (SES-G3), relatively wealthy (SES-G4).

#### Consumption of fish at household level

Overall, 81% (n = 578) of households reported having consumed fish in the preceding 24 hours. Among fish products consumed, fresh fish and dried fish were the most frequently consumed, compared to smoked or salted fish products. In 36.8% (n = 263) of the households, certain household members did not consume fish at all or did not consume specific fish species. Among those household members who did not consume or did not consume specific fish species, a small proportion of children (10.1%, n = 10) did not consume fish, and 50.5% (n = 50) did not consume *Milonge* (*Clarias theodorae*); 36.4% (n = 36) did not consume *Kababa* (*local term for small fish in general*); 11.1% (n = 11) did not eat *Daaga* (*Rastrineobola argentea*), and 9.1% (n = 9) did not eat other small fish species, such as *Kapenta* and *Chisense (Limnothrissa miodan and Stolothrissa miodon*). Of the 360 children aged 6–23 months, the majority (77.2%, n = 278) consumed fish. In this age category, 12.2% made up the largest proportion among those who did not eat fish, whereas, with increase in age, the proportion of children who did not eat fish decreased (Table 6).

**Table 6:** Fish consumption in children aged 6–23 months.

Age (months)	Did not consume fish		Consumed fish		Total
6–8	**n** 44	**%** 12.2	**n** 27	**%** 7.5	**N** 71
9–11	14	3.9	59	16.4	73
12–14	13	3.6	64	17.8	77
15–17	6	1.7	48	13.3	54
18–20	3	0.8	44	12.2	47
21–23	2	0.6	36	10	38
Total	82	22.8	278	77.2	360

With regards to fish consumption among pregnant and lactating mothers, respondents were asked to report whether pregnant and lactating women in their community were allowed to consume fish. Most (90.5%, n = 646), reported that pregnant and lactating women consumed fish, with no significant differences between wealth groups. Respondents gave various reasons that hinder fish consumption by pregnant and lactating women, including: fish gives rashes; unpleasant smell; health concerns due to unhealthy preservatives used; risk of swallowing bones; some fish species are associated with evil spirits; negative effects on the unborn child (e.g. baby being born with ringworm), and fish causes some women to feel sick (e.g. vomiting and nausea).

Analysis of fish consumption by wealth group indicated that more households in SES-G4, (relatively wealthy) reported consumption of fish in the preceding 24 hours compared to those from SES-G1 (poorest). Chi square test indicated a significant difference in fish consumption by wealth group (p = 0.003). Differences in patterns of fish consumption between wealth groups were with respect to the size of fish. Households belonging to SES-G3 and SES-G4 tended to eat significantly more fresh large fish (p = 0.001) or dried large fish (p = 0.016) compared to the lower wealth groups. Significant differences were noted in the frequency of consumption by wealth group of fresh large fish (p = 0.000) and dried large fish (p = 0.043). A high proportion of households in SES-G4 (52.3%) consumed fresh large fish as well as dried large fish (33.1%) more frequently compared to households in the lower wealth groups (Table 7). There were no significant differences observed in the frequency of consumption of fresh small fish, dried small fish and smoked small fish among households from the different wealth groups.

**Table 7:** Fish products consumed at the household level in the preceding seven days.

Fish Product	SES-G1 n (%)	SES-G2 n (%)	SES-G3 n (%)	SES-G4 n (%)	p-value (χ^2^)	Total N (%)
Fresh small fresh fish	35 (20.0)	42 (24.0)	43 (24.9)	55 (28.5)	.*303*	175 (30.3)
Fresh large fish	53 (30.0)	58 (33.1)	84 (49.1)	101 (52.3)	.*000*	296 (51.2)
Dried small fish	70 (40.0)	69 (39.4)	77 (45.0)	72 (37.3)	.*499*	288 (49.8)
Dried large fish	38 (21.7)	40 (22.9)	50 (29.2)	64 (33.1)	.*043*	192 (33.2)
Smoked small fish	6 (3.4)	1 (0.6)	4 (2.3)	4 (2.1)	.*316*	15 (2.6)
Smoked large s fish	4 (2.3)	1 (0.6)	3 (1.8)	2 (1.0)	.*531*	10 (1.7)
Salted Fish	4 (2.3)	3 (1.7)	3 (1.8)	3 (1.6)	.*959*	12 (2.1)

Socio-economic status group (SES-G1-4): Poorest (SES-G1), second (SES-G2), third (SES-G3), and relatively wealthy (SES-G4).

Regardless of the fish species and fish product consumed, the mean intake of fish consumed was 91.4 g/d in women; 36.9 g/d in children aged 6–23 months; 49.0 g/d in children aged 24–59 months; and 110.3 g/d in men. There were differences regarding the quantity of fish consumed among the various wealth groups, but these were not significant. Given that about 80% of the households surveyed consumed fish at least once per week, conservative estimates of annual fish consumption (average fish consumption per meal x 52 weeks) are: 4.8 kg/y in women; 1.9 kg/y in children aged 6–23 months; 2.5 kg/y in children aged 24–59 months; and 5.7 kg/y in men.

### Nutritional status of children and women

The length/height for age Z-scores (HAZ), weight for age Z scores (WAZ) and weight for length/height (WHZ) indices were used to determine the nutritional status of children. The HAZ scores were determined for 359 children aged 6–23 months and 397 children aged 24–59 months. Prevalence of stunting was 37.4% in children aged 6–23 months and 43.3% in children aged 24–59 months; higher than those reported in the Zambia DHS 2013–2014 (35.7% stunting in Lusaka Province) [27,28]. About 25.9% of children aged 24–59 months were moderately stunted. Similar proportions of children aged 6–23 months and 24–59 months were severely stunted (Table 8).

**Table 8:** Length/height for age Z-scores (HAZ), weight for length/height Z-scores (WHZ), and weight for age Z-scores (WAZ) in children aged 6–59 months.

Category	Children aged 6–23 months		Children aged 24–59 months	
**HAZ**	**n**	**%**	**n**	**%**
Normal	225	62.7	225	56.7
Stunted	134	37.4	172	43.3
Moderate stunted	76	21.2	103	25.9
Severely stunted	58	16.2	69	17.4
Total	359	100	397	100
**WHZ**	**n**	**%**	**n**	**%**
Normal	282	78.6	352	88.7
Wasted	25	7	14	3.3
Moderate wasted	16	4.5	7	1.8
Severe wasted	9	2.5	6	1.5
Overweight	52	14.5	32	8.1
Total	359	100	397	100
**WAZ**	**n**	**%**	**n**	**%**
Normal	324	90	342	85.7
Underweight	35	9.7	55	13.9
Moderate underweight	34	9.4	42	10.6
Severe underweight	1	0.3	13	3.3
Total	359	100	397	100

Prevalence of wasting was 7% in children aged 6–23 months and 3.3% in children aged 24–59 months. A higher proportion of children (14.5%) aged 6–23 months was overweight compared to those aged 24–59 months (8.1%), as well as those who suffered from moderate and severe wasting. The prevalence of underweight was 9.7% in children aged 6–23 months and 13.9% in children aged 24–59 months. A t-test showed a significance difference in underweight in the two age groups of children (t = 48.76; df = 758 and p = 0.00). There were no significant differences in the proportions of children aged 6–23 months who were stunted, wasted and underweight by wealth category. A higher proportion of children aged 24–59 months in the SES-G4 wealth group (relatively wealthy), were within the normal range for HAZ, WHZ and WAZ scores. More children from the poorest wealth group, SES-G1 wealth group (poorest) were severely stunted, followed by those in SES-G3 wealth group. There were significant differences in HAZ scores for children aged 24–59 months by different wealth categories (Chi square value = 15.451; df = 6; p = 0.017).

There were no significant differences in WHZ and WAZ scores by wealth groups in this age group. A t-test showed significant differences in HAZ (mean = -1.130 (±2.520), t = -8.499, p = 0.000), WAZ (mean = -0.390 (±1.34), t = -5.505, p = 0.000) and WHZ (mean = 0.264 (±1.653), t = 3.304) between girls and boys aged 6–23 months. The same trend was observed in children aged 24–59 months, with statistical values for HAZ (mean = -1.737 (±1.673), t = -20.69; p = 0.000), WHZ (mean = -0.720 (±4.98), t = 2.261, p = 0.024) and WAZ (mean = -0.720 (±2.609); t = -5.502, *p* = 0.000).

There was a statistically significant difference in nutritional status (as measured by HAZ) in children aged 6–23 months, in relation to fish consumption (χ^2^ = 10.979, df = 2, p = 0.004). A higher proportion of children who consumed fish (45.7%, n = 164) were within the normal range of HAZ score compared to those who did not consume fish. Spearman rank order correlation coefficient indicated a significant correlation between fish consumption and HAZ score (r = 0.139, p = 0.008). There was no statistically significant association between fish consumption and WAZ or WHZ scores.

More than half of the mothers (59.4%, n = 423) were within the normal BMI range (BMI 18.5–25); 22.3% (n = 159) were overweight (BMI ≥25.00–29.9); and 5.5% (n = 39) were underweight (BMI <18.50). Few women (11.8%, n = 84) were found to be obese (BMI ≥30.00), and 1% (n = 7) were found to be morbidly obese (BMI ≥40.00). Mothers’ nutritional status by wealth group showed no significant differences. Spearman rank order correlation coefficient indicated no significant correlation between fish consumption and women’s BMI (rho = -0.042, p = 0.927).

### Dietary diversity and stunting determinants in children aged 6–59 months

A Poisson regression was conducted to determine factors that influence dietary diversity in children. Mothers’ education and child’s age were significant determinants of dietary diversity in children aged 6–23 months. In children aged 24–59 months, wealth status of the household had a significant influence on the child’s dietary diversity (coefficient = 0.827, p = 0.011). Other factors (child’s sex, mother’s occupation, morbidity and age) were not significant at p = 0.05.

Table 9 shows the results of logistic regression models for determinants of stunting in children aged 6–23 months and 24–59 months. The models for the two age groups were estimated separately. The most significant risk factors for stunting in children aged 6–23 months were child’s age (AOR = 0.858 (95% CI: 0.800–0.919), mother’s BMI (AOR = 1.057 (95% CI:1.001–1.117), access to treated water (AOR = 0.496 (95% CI: 0.300–0.820), and child’s morbidity AOR = 0.543 (95% CI:0.256–1.154). The quantity of fish consumed by children was significantly associated with stunting in both age groups; adjusted odds ratio of 0.947 (95% CI: 0.896–1.000) for children aged 6–23 months and adjusted odds ratio of 1.038 (CI: 1.006–1.072) for children aged 24–59 months. In children aged 24–59 months, access to treated water and child’s morbidity were also found to be significantly associated with stunting.

**Table 9:** Regression results of the determinants of stunting in children aged 6–23 months and 24–59 months.

	Children aged 6–23 months		Children aged 24–59 months	
	Odds ratio (95% C.I.)	P	Odds ratio (95% C.I.)	P
Child’s sex (1 = male)	0.921 (0.559–1.515)	0.745	0.761 (0.492–1.177)	0.219
Child’s age in months	0.858 (0.800–0.919)	0.000	1.019 (0.998–1.042)	0.082
Child was ill in the preceding two weeks (1)	0.543 (0.256–1.154)	0.112	0.383 (0.243–0.602)	0.000
Child breastfeeding (1 = yes)	1.054 (0.533–2.086)	0.879	-	-
Quantity of fish consumed (in the preceding 24 hours)	0.947 (0.896–1.000)	0.049	1.038 (1.006–1.072)	0.021
Dietary diversity	0.875 (0.680–1.127)	0.301	0.681 (0.437–1.063)	0.091
Fish consumed by child (1 = yes)	0.949 (0.338–2.670)	0.921	2.052 (0.894–4.710)	0.090
Milk and milk products consumed (in the preceding 24 hours) (1 = yes)	0.723 (0.379–1.381)	0.326	0.781 (0.502–1.217)	0.275
Wealth group				
SES-G1		0.691		0.581
SES-G2	0.693 (0.343–1.400)	0.306	0.752 (.399–1.416)	0.377
SES-G3	0.727 (0.362–1.459)	0.370	0.647 (.348–1.203)	0.169
SES-G4	0.927 (0.462–1.862)	0.832	0.781 (.425–1.434)	0.425
Access to treated water (1 = yes)	0.496 (0.300–0.820)	0.006	0.462 (0.296–0.720)	0.001
Mother’s BMI	1.057 (1.001–1.117)	0.046	1.014 (0.969–1.061)	0.548
Mother’s educational level		0.888		0.287
Primary	.929 (.202–4.278)	0.925	1.749 (0.582–5.254)	0.319
Secondary	1.539 (0.409–5.788)	0.524	1.065 (0.448–2.534)	0.887
Tertiary	.943 (.491–1.810)	0.859	1.672 (0.903–3.096)	0.102
Constant	0.742	0.938	91.839	0.102

## Discussion

This study examined socio-economic determinants of food consumption amongst WRA and children under five years of age from households in urban poor settlements of Lusaka, Zambia and the contribution of fish consumption to their nutritional status. Starchy staples were the most consumed food group by almost all women and children. Among animal-source foods, fish was the most consumed by a high proportion of women and children, whereas, eggs and dairy products were seldom consumed. In recent findings on dietary diversity and animal-source foods consumption at household level in Ethiopia, it was observed that the most commonly consumed food group was cereals, whereas, fish, eggs and fruit were the least consumed [3]. Dietary intake is one of the direct causes of malnutrition among children, according to the UNICEF conceptual framework [37].

Consumption of a diverse diet and animal-source foods are associated with reduced risk of stunting, wasting and underweight in children under five years of age, as reported in studies conducted in Ethiopia, Vietnam and Cambodia [38,39]. The results from this study show that the diet of most children was not diverse, an indication of inadequate micronutrient supply from consumed foods. Younger children, aged 6–23 months, had a lower DDS compared to the older children, aged 24–59 months. A large proportion (87.5%) of women achieved minimum dietary diversity, suggesting a greater likelihood of having adequate micronutrient intakes. There were no notable differences in dietary diversity of women by wealth group, however, the majority of women consumed low nutrient density foods. High consumption of these food items can contribute to women being at risk of overweight/obesity, cardio-vascular diseases and other lifestyle diseases. Unlike in women; in children, a difference in dietary diversity by wealth group was found, which is in consensus with other studies reporting increase in dietary diversity with income and wealth [3,40]. Similar low dietary diversity found in this study has been observed among informal settlement populations[41,42] where despite mothers being overweight/obese, their children were stunted/underweight or obese. It has been debated that this co-existence of malnutrition in mothers and their children is associated with the nutrition transition of increased consumption of energy-dense foods that are not nutrient-dense, hence not providing adequate nutrients, thus leading to malnutrition. In Ethiopia, this phenomenon was noted in both rural and urban settings where intake of animal-source foods was much lower in rural than in the urban settings[41]. The results from the Poisson regression showed that child’s age and mother’s educational level were determinants of dietary diversity in children aged 6–23, whereas, wealth status was a significant determinant of dietary diversity in children aged 6–59 months.

Fish consumption in women and men was below the estimated annual per capita consumption of 18.8 kg/capita/y in developing countries, in 2013 [43]. In 2016, globally, annual per capita fish consumption rose to above 20 kg/capita/y; due to growth in aquaculture systems[43]. Consumption estimates for women in this study are much lower than the global per capita consumption. The consumption of fish in Zambia declined from 12.1 kg/capita/y in 1970s to 6.1 kg/capita/y in 2008 [44] and rose slightly to 6.4 kg/capita/y in 2012 [45]. This drop in fish consumption is attributed to the decline in fish stocks as well as an increase in demand due to the increase in the human population.

A high prevalence of stunting in children aged 24–59 months was found in this study in comparison to that reported for the Lusaka Province in the Zambia DHS 2013–2014 [5]. Stunting among children in poor urban settlements of Lusaka can be attributed to inadequate consumption of animal-source foods and overall low dietary diversity, indicating inadequate micronutrient supply. Low dietary diversity is associated with stunting[39,46] and consumption of a diverse diet is associated with a reduction in stunting [38,39]. A higher proportion of children aged 6–23 months was found to be wasted, compared to the older children aged 24–59 months). A higher proportion of children aged 6–23 months were overweight compared to those aged 24–59 months as well as for those suffering from moderate and severe wasting. In this study, higher proportions of children were found to be overweight/obese than the reported estimates for children in Lusaka Province, according to the Zambia DHS 2013–2014. In Zambia, overall childhood overweight/obesity is reported to be 1% [5]. Overweight/ obesity among children in this study can be attributed to the high intakes of starchy foods, sugary/sweet and fatty foods. Consumption of sugar-sweetened beverages has been found to be a key contributor to overweight/obesity due to high content of added sugar, high energy intake from and low satiety of these beverages [47–49]. A high proportion of children in both age groups consumed low-cost processed foods rich in fats and sugar, high in energy and with no nutritive value. Although physical activity among children was not investigated in this study, low physical activity has been shown to contribute to overweight/obesity among children [9] and may also partly explain our findings, in line with findings from both developing and developed countries that children from poor households were overweight/obese [50,51]. Low physical activity, high unemployment, low educational level, irregular meals and chaotic home environment are cited as contributing factors to overweight/obesity [24,52,53]. In Zambia, the increase in overweight/obesity among the poor can be an indication of changing lifestyles and adoption of less traditional diets, especially in urban settings. Findings in this study indicate that children from poorer households were more likely to be overweight/ obese than those from wealthier households. These findings are inconsistent with earlier results [53], showing that children from wealthier households were more likely to be overweight/obese than those from poorer households. Further research on lifestyle behaviours and the home environment and how these affect child nutritional status among the urban poor in Zambia would be valuable.

Notable differences were also found in HAZ and WAZ by sex, in both age groups of children. Despite the overall low dietary diversity of the child’s diet across wealth groups that could have contributed to the poor child nutritional status, fish consumption by children in the relatively wealthy households had a bearing on the children’s better nutritional status. The findings of this study indicated that children from the third and fourth wealth quartiles (SES-G3 and SES-G4) had higher mean intakes of fish. HAZ was associated with fish consumption. A high proportion of children who consumed fish were found to be within the normal range of HAZ scores. From the results of the logistic regression, the quantity of fish consumed by children pointed to a better nutritional outcome (normal HAZ). Children consumed mainly whole small fish (*Kapenta*). Small fish species found in developing countries have been shown to have high levels of vitamin A, iron and zinc [54] micronutrients which are critical for child growth and development. Fish is also a rich source of vitamin B12, only found in animal-source foods, and which is essential for multiple functions, including growth, brain function and nervous system maintenance [17]. Small fish is an important source of highly bioavailable calcium, and particularly important in the diets of children in poor households of Lusaka.

A high proportion of mothers were found to be overweight/ obese. The high prevalence of overweight among mothers can be attributed to intake of high energy foods (mainly staples) as earlier reported. Among all provinces in Zambia, the highest proportion of overweight/ obesity among women is found in Lusaka Province (35%), and the lowest in Western Province (10%); and is correlated with educational level and wealth status [5]. Overweight/ obesity is associated with high energy intake and a sedentary lifestyle [9]. Although genetic factors may be important in determining nutritional status of women, socio-cultural, socio-economic, environmental and behavioural factors bear strong relation to nutritional status [9].

### Study limitations

The study had some limitations. The cross-sectional nature of the data does not allow examining causality in the relationships between dietary diversity and socio-economic factors, as well as causality of fish consumption and nutritional status.

## Conclusion

Wealth status, mother’s educational level and child’s age had an influence on the dietary diversity of children. Fish was the most consumed animal-source food by both women and children and can therefore contribute towards the much-needed nutrients from animal-source foods; especially in children aged 6–59 months. Small fish (*Kapenta*) is particularly important in the diets of children in poor urban households in Zambia and contributes to better nutritional outcomes. As all small fish stem from capture fisheries, sustainable one health environmental integration, monitoring and management strategies are desirable. Older children aged 24–59 months had a higher prevalence of stunting than those aged 6–23 months. This is attributed to inadequate nutrient adequacy of diets as observed in the low dietary diversity. Furthermore, there are factors that affect stunting that are age-related (e.g. feeding practices and other child care practices). Among the urban poor, both undernutrition, overweight and obesity among children and women are major problems that need a more focused attention by policy makers. The government and development partners should not only channel efforts to solving the stunting problem, but should also implement prevention programmes for overweight/ obesity which is on the increase, especially among children. Utilization of locally produced food in complementary feeding and ensuring that diets are diversified, combined with caring practices, education of mothers and proper sanitary environments are paramount to ensure optimal child nutrition.

## Supporting information

S1 File The S1_File.pdf is the questionnaire that was used to collect data. (PDF)
