# Supplementary material for: Dietary diversity determinants and contribution of fish to maternal and under-five nutritional status in Zambia
Source: PLoS One. 2018 Sep 24;13(9):e0204009. doi: 10.1371/journal.pone.0204009 (PMC6152956; doi:10.1371/journal.pone.0204009)
Supplement: S1 File — (PDF) [file pone.0204009.s001.pdf]

GIZ_HH_Consumption_Survey

SAVE

Layout

Search Library

METADATA:START TIME, END TIME, TODAY, DEVICE ID, SUBSCRIBER ID, PHONE NUMBER

General Information

123 * i. Questionnaire number

abc * ii. Name of enumerator

* iii. Name of supervisor

MIYOBA NIXON	Value:	1
MULONGWE MCDONALD	Value:	2
OLIVER MALUPANDE	Value:	3
CONSTANCE NG'WANE	Value:	4

* iv. Date of survey

v. Take the GPS coordinates of the place!

* vi. Is there a child in the household that is between 2 and 5 years of age?

Yes	Value:	1

No Value: 0

* vii. Is there any infant in the household that is aged between 6-23 months?

Yes Value: 1

No Value: 0

* viii. Consent: the respondent agreed to provide information?

Yes Value: 1

No Value: 0

This household does not fulfill the eligibility criteria! Household needs to be replaced!

abc * If no, please reason why not!

Section A: Identification Particulars of a Household

abc Name of respondent? (optional)

* Name of constituency:

Kanyama	Value: kanyama
Matero	Value: matero
Munali	Value: munali

* Name of ward:

Kanyama	Value: kanyama
Matero	Value: matero
Mtendere	Value: mtendere

* CSA:

2	Value: 2
14	Value: 14
16	Value: 16
17	Value: 17
19	Value: 19
22	Value: 22
23	Value: 23
25	Value: 25
26	Value: 26
30	Value: 30
32	Value: 32
53	Value: 53

54	Value: 54
55	Value: 55
12	Value: 12
13	Value: 13
14	Value: 14
3	Value: 3
4	Value: 4
11	Value: 11
15	Value: 15
17	Value: 17
18	Value: 18
19	Value: 19
20	Value: 20
21	Value: 21
22	Value: 22
24	Value: 24
25	Value: 25
26	Value: 26
30	Value: 30
32	Value: 32

* SEA:

3	Value: 3
2	Value: 2
1	Value: 1

5	Value:	5
3	Value:	3
3	Value:	3
5	Value:	5
1	Value:	1
2	Value:	2
4	Value:	4
1	Value:	1
1	Value:	1
1	Value:	1
5	Value:	5
4	Value:	4
1	Value:	1
2	Value:	2
3	Value:	3
4	Value:	4
5	Value:	5
1	Value:	1
2	Value:	2
3	Value:	3
4	Value:	4
5	Value:	5
6	Value:	6
1	Value:	1
2	Value:	2
3	Value:	3
1	Value:	1
4	Value:	4

1	Value: 1
4	Value: 4
5	Value: 5
5	Value: 5
2	Value: 2
5	Value: 5
1	Value: 1
4	Value: 4
1	Value: 1
3	Value: 3
3	Value: 3

* Ethnic group

Lozi	Value: 1
Mbunda	Value: 2
Luvale	Value: 3
Kaonde	Value: 4
Bemba	Value: 5
Tonga	Value: 6
Other (please specify!)	Value: 998

abc * If other, please specify!

Section B: Demographic and Social Economic Information

123	* When were you born?
1+1	int(2015 - ${B1a})
≡	Respondent is ${B1b} years old.
⊙	* Indicate the gender:

Option	Value
Female	1
Male	0

⊙ * What is the relationship to the child?

Option	Value
Father	1
Mother	2
Sister	3
Brother	4
Uncle	5
Aunt	6
Grandmother	7
Grandfather	8
Other (please specify!)	998

abc * If other, please specify!

* What is the highest level of education you have attained?

None	Value: 0
Primary (Grade 1-4)	Value: 1
Primary (Grade 5-7)	Value: 2
Secondary (Grade 8-9)	Value: 3
Secondary (Grade 10-12)	Value: 4
Tertiary	Value: 5

* What is your marital status?

Single	Value: 1
Married	Value: 2
Cohabiting	Value: 3
Separated	Value: 4
Divorced	Value: 5
Widowed	Value: 6

123 * How many people live in this household?

123 * How many family members are below the age of 5 years in your household?

* Are you in formal or informal employment?

Formal	Value: 1
Informal	Value: 2
Unpaid family member	Value: 3

* Specify the types of employment a bit more, please!

Tailor	Value: 1
Shopkeeper	Value: 2
Fish monger	Value: 3
Teacher	Value: 4
Business	Value: 5
Other (please specify!)	Value: 998

abc If other, please specify!

Income activities

What are the top 3 main income generating activities for your household?

* new question

Salaried employment	Value: 1
Doing piecework (manual work)	Value: 2
Running small business (sales of veggies, rice, maize, beans, etc.)	Value: 3
Runnng small business (small shops, stationary, etc)	Value: 4
Firewood / charcoal sales	Value: 5
Skilled trade / artisan activity (carpenter, blacksmith, mason)	Value: 6
None	Value: 7
Other (please specify!)	Value: 998

abc * If other, please specify!

* new question

Salaried employment	Value: 1
Doing piecework (manual work)	Value: 2
Running small business (sales of veggies, rice, maize, beans, etc.)	Value: 3
Runnng small business (small shops, stationary, etc)	Value: 4
Firewood / charcoal sales	Value: 5
Skilled trade / artisan activity (carpenter, blacksmith, mason)	Value: 6
None	Value: 7
Other (please specify!)	Value: 998

abc * If other, please specify!

* new question

Salaried employment	Value: 1
Doing piecework (manual work)	Value: 2
Running small business (sales of veggies, rice, maize, beans, etc.)	Value: 3
Runnng small business (small shops, stationary, etc)	Value: 4
Firewood / charcoal sales	Value: 5
Skilled trade / artisan activity (carpenter, blacksmith, mason)	Value: 6
None	Value: 7
Other (please specify!)	Value: 998

abc * If other, please specify!

Characteristics of housing

* What is the main construction material of the walls of your house?

Cane / palm / trunks	Value: 1
Mud	Value: 2
Bamboo / pole with mud	Value: 3
Stone with mud	Value: 4
Plywood	Value: 5
Cardboard	Value: 6
Reused wood	Value: 7
Cement	Value: 8

Stone with lime / cement	Value:	9
Bricks	Value:	10
Cement blocks	Value:	11
Wood planks	Value:	12
Other (please specify!)	Value:	998

abc * If other, please specify!

* What material is the roof made of?

Thatch / palm leaf	Value:	1
Rustic mat	Value:	2
Palm / bamboo	Value:	3
Wood planks	Value:	4
Cardboard	Value:	5
Metal / iron sheets	Value:	6
Wood	Value:	7
Calamine / cement fibre (Asbestos)	Value:	8
Ceramic tiles / Harvey tiles	Value:	9
Cement	Value:	10
Roofing shingles	Value:	11
Mud tiles	Value:	12
Other (please specify!)	Value:	998

abc * If other, please specify!

* What material is the floor of the house made of?

Option	Value
Earth / sand	1
Dung	2
Wood planks	3
Palm / bamboo / leeds	4
Parquet or polished wood	5
Concrete cement	6
Tiles	7
Vinyl (PVC) or asphalt strips	8
Carpet	9
Other (please specify!)	998

abc * If other, please specify!

* How many rooms does your main house have?

Option	Value
One	1
Two	2
Three or more	3

123 * How many rooms in this household are used for sleeping?

Water and sanitation

* What is your main source of drinking water?

Option	Value
Piped into Dwelling	1
Piped into Yard/Plot	2
Public taps / standpipe	3
Protected dwell	4
Unprotected dwell	5
Protected spring	6
Unprotected spring	7
Rainwater	8
Tanker Truck	9
Cart with small tank	10
Surface water (river, pond, canal, stream)	11
Bottled water	12
Other (please specify!)	998

abc

* If other, please specify!

* What do you usually do to make the water safer to drink?

Option	Value
Boil	1
Add Bleach / chlorine / clorin	2
Strain through a cloth	3
Use water filter	4
Solar disenfection	5

Let it stand and settle	Value: 6
Nothing	Value: 7
Other (please specify!)	Value: 998

abc * If other, please specify!

* How do you store your drinking water?

Closed container / jerry can	Value: 1
Open container / bucket	Value: 2
Does not store water	Value: 3
Other (please specify!)	Value: 998

abc * If other, please specify!

* What kind of toilet facility do members of your household use?

Flush or pour flush toilet (to piped sewer system)	Value: 1
Flush to septic tank	Value: 2
Flush to pit latrine	Value: 3
Flush to somewhere else	Value: 4
Flush to don't know where	Value: 5
VIP latrine	Value: 6
Pit latrine with slab	Value: 7

Pit latrine without slab / open pit	Value: 8
Composing Toilet	Value: 9
Bucket toilet	Value: 10
Hanging toilet / latrine	Value: 11
No facility / bush / field	Value: 12
Other (please specify!)	Value: 998

abc * If other, please specify!

Assets owned by the household

* Bicycles

Yes	Value: 1
No	Value: 0

* Motorcycle

Yes	Value: 1
No	Value: 0

* Car

Yes Value: 1

No Value: 0

* Solar panels

Yes Value: 1

No Value: 0

* Car batteries

Yes Value: 1

No Value: 0

* Television (TV)

Yes Value: 1

No Value: 0

* Radio

Yes Value: 1

No Value: 0

* Watch or clock

Yes	Value:	1
No	Value:	0

* Rental houses

Yes	Value:	1
No	Value:	0

* Wheelbarrow

Yes	Value:	1
No	Value:	0

* Axe

Yes	Value:	1
No	Value:	0

* Refrigerator

Yes	Value: 1
No	Value: 0

Further assets owned by the household

* Mobile phone

None	Value: 0
One	Value: 1
More than one	Value: 2

* Cultivatable land

Yes	Value: 1
No	Value: 0

1.0 * How much land (ha) do you own?

Section C: The 24 Hour Recall

Before Beginning The 24 Hour Recall: ENUMERATOR, ask the respondent if the previous 24 hour period was "usual" or "normal" for the his/her household. If there was a special occasion, such as a funeral or feast, another day should be selected for the interview. If this is not possible, please select another household instead of asking the respondent about an earlier day in the week.

Day of the week:

* What day is it today?

Monday	Value: 1
Tuesday	Value: 2
Wednesday	Value: 3
Thursday	Value: 4
Friday	Value: 5
Saturday	Value: 6
Sunday	Value: 7

Personal Details:

abc * What is your name?

The 24 Hour Recall (mother or permanent care taker of the children)

ENUMERATOR: Find out all the food and drinks (including drinking water) ${name} ate since waking up yesterday until ${name} went to sleep the next day. This includes snacks and drinks consumed between meals. Steps: \n1. Ask ${name} about the first meal he/she had yesterday. \n2. Ask ${name} about all the food and drinks he/she had during that meal. Write only ONE food or drink in each section A box. Click "+" on the lower right corner of section A to add a new box for each food or drink. Remember to ask about quantity of water added when cooking. 3. If there is a mixed dish, please ask ${name} for a list and quantities of ingredients. Remember to probe for added foods such as sugar in tea, oil in mixed dishes and fried food. Write each ingredient in a new section A box. Do not write down the mixed dish. ONLY write down the ingredients. \n4. After ${name} recalls all the foods and drinks consumed for that meal, probe him/her to ensure that there is nothing else that he/she forgot to mention. If yes, add a new section A box and write in it. \n5. Probe ${name} until he/she says “nothing else” before moving to the next meal. \n6. Ask ${name} about the next meal he/she had yesterday. \n7. Repeat steps 2-6 until ${name} says that he/she went to sleep the next day. 8. Then go back and complete field C, D and E for each food item.

Questions:

* B. What did you eat or drink?

Avocado fresh	Value: F502111
Banana Ripe	Value: 1
Beans Solwezi Z	Value: Z000021
Beans. Kabulangeti Z	Value: Z000020
Beans. Kidney.boiled without salt	Value: Z000023
Beans. white. boiled W	Value: Z000019
Beef fat fresh cooked	Value: U103121
Beef medium fat fresh cooked	Value: U102121
Beef stomach/tripe cooked (offals)	Value: V551021
Biscuits	Value: D700000
Boiled potatoes (R)	Value: _193301
Bondwe boiled without salt W	Value: Z000011
Bream (fish)	Value: Z000034
Brown bread from wheaten mixture	Value: B251000
Busala	Value: 2
Carrot fresh	Value: G620111
Carrot fresh cooked	Value: G620121
cassava leaves fresh cooked with groundnuts	Value: Z000049
caterpillars (vinkhulubala)	Value: 3
Chicken	Value: 21
Chikanda	Value: 4
Chinese cabbage	Value: 20
Chinese cabbage fresh cooked	Value: G321121

Chips (French-fried potatoes) (R)	Value:	_164700
Cola beverages (caffeinic)	Value:	N330000
Corn puffs	Value:	22
Cow's milk boiled	Value:	M110030
Dairy Products-Tinned or Fresh milk	Value:	5
Doughnuts (R)	Value:	_115001
Eggs Boiled	Value:	Z000031
Fish small dried with oil and tomatoes	Value:	Z000008
Fried sausage (R)	Value:	_025002
Groundnuts. Dried. raw Z	Value:	Z000015
Groundnuts. Powder T	Value:	Z000017
Impwa	Value:	18
Inswa	Value:	6
Kapenta (fish)	Value:	Z000018
Lumanda	Value:	7
Maize breakfast meal Z	Value:	Z000025
Maize Porridge with sugar	Value:	Z000046
Maize roller meal Z	Value:	Z000026
Maize samp	Value:	8
Mango fresh	Value:	F516111
Manioc (cassava)	Value:	K410000
Mice (mbeba)	Value:	9
Munkoyo	Value:	10
Mushrooms fresh cooked	Value:	K700121
Natural youghurt	Value:	11
Nshima Maize	Value:	Z000022
Nshima, maize and sorghum	Value:	Z000029
Nshima, sorgum and millet	Value:	Z000030

Okra Lady fingers	Value: 12
Onion	Value: 19
Onions fresh cooked	Value: G480121
Orange fresh	Value: F603111
Papaya fresh	Value: F517111
Peanut butter	Value: 13
Pizza	Value: _164100
Pumpkin leaves cooked with groundnuts	Value: Z000063
Pumpkin leaves relish without oil	Value: Z000006
Rape fresh boiled	Value: Z000010
Rice parboiled cooked	Value: C359022
Salt	Value: 14
Shake and Sip (maheu)	Value: Z000040
Sour milk	Value: 15
Sugar T	Value: Z000012
Sunflower oil	Value: 16
Sweet potato fresh EP	Value: Z000038
Sweet potato leaves boiled	Value: Z000003
Sweet potato leaves cooked with groundnuts	Value: Z000052
Tomato red fresh	Value: G561111
Tomatoes fresh cooked	Value: G560121
Water	Value: 17
Watermelon fresh	Value: F509111
Wholemeal bread	Value: B101000
Other (please specify!)	Value: 998

abc

* If OTHER, please specify:

Please complete Sections: C, D and E ONLY after you have written down the all the food and drinks ${name} ate since waking up yesterday until ${name} went to sleep the next day.

* C. How was this food item prepared?

Raw / fresh	Value: 1
Boiled	Value: 2
Cooked	Value: 3
Roasted	Value: 4
Fried	Value: 5
Steamed	Value: 6
Baked	Value: 7
Smoked	Value: 8
Dried	Value: 9
Processed	Value: 10
Not applicable	Value: 999
No answer	Value: 997

1.0 * D. Amount / quantity of food/drink consumed:

* E. Unit of measurement:

g (grams)	Value: 1
ml (milliliters)	Value: 2

* F. Category:

Group 1A: Grains , roots, and tubers. i.e. maize meal porridge, maize meal and cassava porridge, sorghum and millet porridge, bread, rice, munkoyo and other foods made from grains.	Value:	1a
Group 1B: White potatoes, sweet potatoes, white yams, cassava, chikanda and other foods made from roots.	Value:	1b
Food item was not mentioned in or does not fit any of the food categories above.	Value:	10
Group 2: Legumes and nuts. i.e. beans, ground nuts, peas, lentils and seeds.	Value:	2
Group 3: Milk and milk products (e.g.: cheese, milk (fresh & processed), sour milk, yoghurt, etc.)	Value:	3a
Group 4: Flesh foods. i.e. organ meats (Liver, Kidney, Heart, Tongue) and any meat (Beef, Pork, Lamb, Goat, Chicken or Duck).	Value:	4
Group 5: Eggs	Value:	5
Group 6A: Vitamin A rich fruit and vegetables yellow/orange colour in the inside (e.g. papaya, pumpkin, carrots, squash, or orange-fleshed sweet potatoes, guava, mangoes)	Value:	6a
Group 6B: Vitamin A rich vegetables. i.e. dark green vegetables, bondwe, chibwabwa, kalembula, Chinese cabbage	Value:	6b
Group 6C: other vegetables e.g. impwa, cucumber, cabbage	Value:	6d
Group 7: Other fruit and vegetables. i.e. wild fruit (Mfungo, Mutungulu, Chisongole) or any other wild fruit or vegetables.	Value:	7
Group 8A: Others (i.e. any oil, fats, or butter or foods made with any of these)	Value:	8
Group 8B: Others (i.e. any sugary foods (Chocolates, Sweets, Candies, Pastries, Cakes or Biscuits))	Value:	8b
Group 8C: Others (i.e. condiments for flavor (Chillies, Spices, Herbs, or Fish powder))	Value:	8c
Group 9: Beverages (i.e. tea, coffee, etc.)	Value:	9

1+1 count(${questions})

Number of food items ${name} ate yesterday: ${C2_5}

24 Hour Recall (child aged 6-23 months)

Identify ONE representative infant from the household and complete the following fields:

* Select gender:

Male	Value: 1
Female	Value: 2

123 * Age in months:

abc * What is her / his name?

Beginning The 24 Hour Recall:

ENUMERATOR: Find out all the food and drinks (including drinking water) ${name_infant} ate since waking up yesterday until the infant went to sleep the next day. This includes snacks and drinks consumed between meals. Steps: \n1. Ask ${name} about the first meal ${name_infant} had yesterday. \n2. Ask ${name} about all the food and drinks ${name_infant} had during that meal. Write only ONE food or drink in each section A box. Click "+" on the lower right corner of section A to add a new box for each food or drink. Remember to ask about quantity of water added when cooking. 3. If there is a mixed dish, please ask ${name} for a list and quantities of ingredients. Remember to probe for added foods such as sugar in tea, oil in mixed dishes and fried food. Write each ingredient in a new section A box. Do not write down the mixed dish. ONLY write down the ingredients. \n4. After ${name} recalls all the foods and drinks ${name_infant} consumed for that meal, probe him/her to ensure that there is nothing else that he/she forgot to mention. If yes, add a new section A box and write in it. \n5. Probe ${name} until he/she says “nothing else” before moving to the next meal. \n6. Ask ${name} about the next meal the ${name_infant} had yesterday.\n7. Repeat steps 2-6 until ${name} says that the ${name_infant} went to sleep the next day. 8. Then go back and complete field C, D and E for each food item.

Questions:

* B. What did ${name_infant} eat?

Avocado fresh	Value: F502111
Banana Ripe	Value: 1
Beans Solwezi Z	Value: Z000021

Beans. Kabulangeti Z	Value:	Z000020
Beans. Kidney.boiled without salt	Value:	Z000023
Beans. white. boiled W	Value:	Z000019
Beef fat fresh cooked	Value:	U103121
Beef medium fat fresh cooked	Value:	U102121
Beef stomach/tripe cooked (offals)	Value:	V551021
Biscuits	Value:	D700000
Boiled potatoes (R)	Value:	_193301
Bondwe boiled without salt W	Value:	Z000011
Bream (fish)	Value:	Z000034
Brown bread from wheaten mixture	Value:	B251000
Busala	Value:	2
Carrot fresh	Value:	G620111
Carrot fresh cooked	Value:	G620121
cassava leaves fresh cooked with groundnuts	Value:	Z000049
caterpillars (vinkhulubala)	Value:	3
Chicken	Value:	21
Chikanda	Value:	4
Chinese cabbage	Value:	20
Chinese cabbage fresh cooked	Value:	G321121
Chips (French-fried potatoes) (R)	Value:	_164700
Cola beverages (caffeinic)	Value:	N330000
Corn puffs	Value:	22
Cow's milk boiled	Value:	M110030
Dairy Products-Tinned or Fresh milk	Value:	5
Doughnuts (R)	Value:	_115001
Eggs Boiled	Value:	Z000031
Fish small dried with oil and tomatoes	Value:	Z000008

Fried sausage (R)	Value:	_025002
Groundnuts. Dried. raw Z	Value:	Z000015
Groundnuts. Powder T	Value:	Z000017
Impwa	Value:	18
Inswa	Value:	6
Kapenta (fish)	Value:	Z000018
Lumanda	Value:	7
Maize breakfast meal Z	Value:	Z000025
Maize Porridge with sugar	Value:	Z000046
Maize roller meal Z	Value:	Z000026
Maize samp	Value:	8
Mango fresh	Value:	F516111
Manioc (cassava)	Value:	K410000
Mice (mbeba)	Value:	9
Munkoyo	Value:	10
Mushrooms fresh cooked	Value:	K700121
Natural youghurt	Value:	11
Nshima Maize	Value:	Z000022
Nshima, maize and sorghum	Value:	Z000029
Nshima, sorgum and millet	Value:	Z000030
Okra Lady fingers	Value:	12
Onion	Value:	19
Onions fresh cooked	Value:	G480121
Orange fresh	Value:	F603111
Papaya fresh	Value:	F517111
Peanut butter	Value:	13
Pizza	Value:	_164100
Pumpkin leaves cooked with groundnuts	Value:	Z000063

Pumpkin leaves relish without oil	Value:	Z000006
Rape fresh boiled	Value:	Z000010
Rice parboiled cooked	Value:	C359022
Salt	Value:	14
Shake and Sip (maheu)	Value:	Z000040
Sour milk	Value:	15
Sugar T	Value:	Z000012
Sunflower oil	Value:	16
Sweet potato fresh EP	Value:	Z000038
Sweet potato leaves boiled	Value:	Z000003
Sweet potato leaves cooked with groundnuts	Value:	Z000052
Tomato red fresh	Value:	G561111
Tomatoes fresh cooked	Value:	G560121
Water	Value:	17
Watermelon fresh	Value:	F509111
Wholemeal bread	Value:	B101000
Other (please specify!)	Value:	998

abc

* If OTHER, please specify:

Please complete Sections: C, D and E ONLY after you have written down the all the food and drinks ${name_infant} ate since waking up yesterday until ${name_infant} went to sleep the next day.

* C. How was this food item prepared?

Raw / fresh	Value:	1

Boiled	Value: 2
Cooked	Value: 3
Roasted	Value: 4
Fried	Value: 5
Steamed	Value: 6
Baked	Value: 7
Smoked	Value: 8
Dried	Value: 9
Processed	Value: 10
Not applicable	Value: 999
No answer	Value: 997

1.0 * D. Amount / quantity of food/drink consumed:

* E. Unit of measurement:

g (grams)	Value: 1
ml (milliliters)	Value: 2

* E. Category:

Group 1A: Grains , roots, and tubers. i.e. maize meal porridge, maize meal and cassava porridge, sorghum and millet porridge, bread, rice, munkoyo and other foods made from grains.	Value: 1a
Group 1B: White potatoes, sweet potatoes, white yams, cassava, chikanda and other foods made from roots.	Value: 1b
Food item was not mentioned in or does not fit any of the food categories above.	Value: 10

Group 2: Legumes and nuts. i.e. beans, ground nuts, peas, lentils and seeds.	Value: 2
Group 3: Milk and milk products (e.g.: cheese, milk (fresh & processed), sour milk, yoghurt, etc.)	Value: 3a
Group 4: Flesh foods. i.e. organ meats (Liver, Kidney, Heart, Tongue) and any meat (Beef, Pork, Lamb, Goat, Chicken or Duck).	Value: 4
Group 5: Eggs	Value: 5
Group 6A: Vitamin A rich fruit and vegetables yellow/orange colour in the inside (e.g. papaya, pumpkin, carrots, squash, or orange-fleshed sweet potatoes, guava, mangoes)	Value: 6a
Group 6B: Vitamin A rich vegetables. i.e. dark green vegetables, bondwe, chibwabwa, kalembula, Chinese cabbage	Value: 6b
Group 6C: other vegetables e.g. impwa, cucumber, cabbage	Value: 6d
Group 7: Other fruit and vegetables. i.e. wild fruit (Mfungo, Mutungulu, Chisongole) or any other wild fruit or vegetables.	Value: 7
Group 8A: Others (i.e. any oil, fats, or butter or foods made with any of these)	Value: 8
Group 8B: Others (i.e. any sugary foods (Chocolates, Sweets, Candies, Pastries, Cakes or Biscuits))	Value: 8b
Group 8C: Others (i.e. condiments for flavor (Chillies, Spices, Herbs, or Fish powder))	Value: 8c
Group 9: Beverages (i.e. tea, coffee, etc.)	Value: 9

1+1 count(${questions_infant})

Number of food items ${name_infant} ate yesterday: ${C3_7}

Infant feeding

* Does ${name_infant} eat fish?

Yes	Value: 1
No	Value: 0

123 * How old was ${name_infant} when you started feeding him / her fish?

* Was ${name_infant} breastfed or did ${name_infant} consume breastmilk yesterday during the day or at night?

Yes	Value:	1
No	Value:	0

123 * How many times did ${name_infant} breast feed yesterday?

24 Hour Recall (child aged 2-5 years)

If yes, identify ONE representative child from the household and complete the following fields:

* Select gender:

Male	Value:	1
Female	Value:	2

123 * Age in months:

abc * What is her / his name?

Beginning The 24 Hour Recall:

ENUMERATOR: Find out all the food and drinks (including drinking water) ${name_child} ate since waking up yesterday until the child went to sleep the next day. This includes snacks and drinks consumed between meals. Steps: \n1. Ask ${name} about the first meal ${name_child} had yesterday. What time was it? \n2. Ask ${name} about all the food and drinks ${name_child} had during that meal. Write only ONE food or drink in each section A box. Click "+" on the lower right corner of section A to add a new box for each food or drink. Remember to ask about quantity of water added when cooking. 3. If there is a mixed dish, please ask ${name} for a list and quantities of ingredients. Remember to probe for added foods such as sugar in tea, oil in mixed dishes and fried food. Write each ingredient in a new section A box. Do not write down the mixed dish. ONLY write down the ingredients. \n4. After ${name} recalls all the foods and drinks ${name_child} consumed for that meal, probe him/her to ensure that there is nothing else that he/she forgot to mention. If yes, add a new section A box and write in it. \n5. Probe ${name} until she says “nothing else” before moving to the next meal. \n6. Ask ${name} about the next meal ${name_child} had yesterday. \n7. Repeat steps 2-6 until ${name} says that ${name_child} went to sleep the next day. 8. Then go back and complete field C, D and E for each food item.

Questions:

* B. What did ${name_child} eat?

Avocado fresh	Value:	F502111
Banana Ripe	Value:	1
Beans Solwezi Z	Value:	Z000021
Beans. Kabulangeti Z	Value:	Z000020
Beans. Kidney.boiled without salt	Value:	Z000023
Beans. white. boiled W	Value:	Z000019
Beef fat fresh cooked	Value:	U103121
Beef medium fat fresh cooked	Value:	U102121
Beef stomach/tripe cooked (offals)	Value:	V551021
Biscuits	Value:	D700000
Boiled potatoes (R)	Value:	_193301
Bondwe boiled without salt W	Value:	Z000011
Bream (fish)	Value:	Z000034
Brown bread from wheaten mixture	Value:	B251000
Busala	Value:	2
Carrot fresh	Value:	G620111
Carrot fresh cooked	Value:	G620121
cassava leaves fresh cooked with groundnuts	Value:	Z000049

caterpillars (vinkhulubala)	Value:	3
Chicken	Value:	21
Chikanda	Value:	4
Chinese cabbage	Value:	20
Chinese cabbage fresh cooked	Value:	G321121
Chips (French-fried potatoes) (R)	Value:	_164700
Cola beverages (caffeinic)	Value:	N330000
Corn puffs	Value:	22
Cow's milk boiled	Value:	M110030
Dairy Products-Tinned or Fresh milk	Value:	5
Doughnuts (R)	Value:	_115001
Eggs Boiled	Value:	Z000031
Fish small dried with oil and tomatoes	Value:	Z000008
Fried sausage (R)	Value:	_025002
Groundnuts. Dried. raw Z	Value:	Z000015
Groundnuts. Powder T	Value:	Z000017
Impwa	Value:	18
Inswa	Value:	6
Kapenta (fish)	Value:	Z000018
Lumanda	Value:	7
Maize breakfast meal Z	Value:	Z000025
Maize Porridge with sugar	Value:	Z000046
Maize roller meal Z	Value:	Z000026
Maize samp	Value:	8
Mango fresh	Value:	F516111
Manioc (cassava)	Value:	K410000
Mice (mbeba)	Value:	9
Munkoyo	Value:	10

Mushrooms fresh cooked	Value:	K700121
Natural youghurt	Value:	11
Nshima Maize	Value:	Z000022
Nshima, maize and sorghum	Value:	Z000029
Nshima, sorgum and millet	Value:	Z000030
Okra Lady fingers	Value:	12
Onion	Value:	19
Onions fresh cooked	Value:	G480121
Orange fresh	Value:	F603111
Papaya fresh	Value:	F517111
Peanut butter	Value:	13
Pizza	Value:	_164100
Pumpkin leaves cooked with groundnuts	Value:	Z000063
Pumpkin leaves relish without oil	Value:	Z000006
Rape fresh boiled	Value:	Z000010
Rice parboiled cooked	Value:	C359022
Salt	Value:	14
Shake and Sip (maheu)	Value:	Z000040
Sour milk	Value:	15
Sugar T	Value:	Z000012
Sunflower oil	Value:	16
Sweet potato fresh EP	Value:	Z000038
Sweet potato leaves boiled	Value:	Z000003
Sweet potato leaves cooked with groundnuts	Value:	Z000052
Tomato red fresh	Value:	G561111
Tomatoes fresh cooked	Value:	G560121
Water	Value:	17
Watermelon fresh	Value:	F509111

Wholemeal bread	Value: B101000
Other (please specify!)	Value: 998

abc

* If OTHER, please specify:

Please complete Sections: C, D and E ONLY after you have written down the all the food and drinks ${name_child} ate since waking up yesterday until ${name_child} went to sleep the next day.

* C. How was this food item prepared?

Raw / fresh	Value: 1
Boiled	Value: 2
Cooked	Value: 3
Roasted	Value: 4
Fried	Value: 5
Steamed	Value: 6
Baked	Value: 7
Smoked	Value: 8
Dried	Value: 9
Processed	Value: 10
Not applicable	Value: 999
No answer	Value: 997

1.0

* D. Amount / quantity of food/drink consumed:

* E. Unit of measurement:

g (grams)	Value: 1
ml (milliliters)	Value: 2

* E. Category:

Group 1A: Grains , roots, and tubers. i.e. maize meal porridge, maize meal and cassava porridge, sorghum and millet porridge, bread, rice, munkoyo and other foods made from grains.	Value: 1a
Group 1B: White potatoes, sweet potatoes, white yams, cassava, chikanda and other foods made from roots.	Value: 1b
Food item was not mentioned in or does not fit any of the food categories above.	Value: 10
Group 2: Legumes and nuts. i.e. beans, ground nuts, peas, lentils and seeds.	Value: 2
Group 3: Milk and milk products (e.g.: cheese, milk (fresh & processed), sour milk, yoghurt, etc.)	Value: 3a
Group 4: Flesh foods. i.e. organ meats (Liver, Kidney, Heart, Tongue) and any meat (Beef, Pork, Lamb, Goat, Chicken or Duck).	Value: 4
Group 5: Eggs	Value: 5
Group 6A: Vitamin A rich fruit and vegetables yellow/orange colour in the inside (e.g. papaya, pumpkin, carrots, squash, or orange-fleshed sweet potatoes, guava, mangoes)	Value: 6a
Group 6B: Vitamin A rich vegetables. i.e. dark green vegetables, bondwe, chibwabwa, kalembula, Chinese cabbage	Value: 6b
Group 6C: other vegetables e.g. impwa, cucumber, cabbage	Value: 6d
Group 7: Other fruit and vegetables. i.e. wild fruit (Mfungo, Mutungulu, Chisongole) or any other wild fruit or vegetables.	Value: 7
Group 8A: Others (i.e. any oil, fats, or butter or foods made with any of these)	Value: 8
Group 8B: Others (i.e. any sugary foods (Chocolates, Sweets, Candies, Pastries, Cakes or Biscuits))	Value: 8b
Group 8C: Others (i.e. condiments for flavor (Chillies, Spices, Herbs, or Fish powder))	Value: 8c
Group 9: Beverages (i.e. tea, coffee, etc.)	Value: 9

1+1 count(${questions_child})

Number of food items ${name_child} ate yesterday: ${C4_7}

24 Hour Recall (male hh member)

* A. Is there any male household member older than 15 years of age?

Yes	Value:	1
No	Value:	0

If yes, identify ONE representative male household member from the household and administer the the following questions directly to him!

* What is your relationship to the child for whom the recall was done before?

Bother	Value:	1
Father	Value:	2
Uncle	Value:	3
Grandpa	Value:	4
Friend of the family	Value:	5
Other (please specify!)	Value:	998

abc * If OTHER, please specify:

123 * Age in years:

abc * What is your name?

Beginning The 24 Hour Recall:

ENUMERATOR: Find out all the food and drinks (including drinking water) ${name_malehh} member ate since waking up yesterday until he went to sleep the next day. This includes snacks and drinks consumed between meals. Steps: \n1. Ask ${name_malehh} about the first meal he had yesterday. \n2. Ask ${name_malehh} about all the food and drinks he had during that meal. Write only ONE food or drink in each section A box. Click "+" on the lower right corner of section A to add a new box for each food or drink. Remember to ask about quantity of water added when cooking. 3. If there is a mixed dish, please ask ${name_malehh} for a list and quantities of ingredients. Remember to probe for added foods such as sugar in tea, oil in mixed dishes and fried food. Write each ingredient in a new section A box. Do not write down the mixed dish. ONLY write down the ingredients. \n4. After ${name_malehh} recalls all the foods and drinks he had consumed for that meal, probe him/her to ensure that there is nothing else that he forgot to mention. If yes, add a new section A box and write in it. \n5. Probe ${name_malehh} until he says “nothing else” before moving to the next meal. \n6. Ask ${name_malehh} about the next meal he had yesterday. \n7. Repeat steps 2-6 until ${name_malehh} says that he went to sleep the next day. 8. Then go back and complete field C, D and E for each food item.

Questions:

* B. What did ${name} eat?

Avocado fresh	Value:	F502111
Banana Ripe	Value:	1
Beans Solwezi Z	Value:	Z000021
Beans. Kabulangeti Z	Value:	Z000020
Beans. Kidney.boiled without salt	Value:	Z000023
Beans. white. boiled W	Value:	Z000019
Beef fat fresh cooked	Value:	U103121
Beef medium fat fresh cooked	Value:	U102121
Beef stomach/tripe cooked (offals)	Value:	V551021
Biscuits	Value:	D700000
Boiled potatoes (R)	Value:	_193301

Bondwe boiled without salt W	Value:	Z000011
Bream (fish)	Value:	Z000034
Brown bread from wheaten mixture	Value:	B251000
Busala	Value:	2
Carrot fresh	Value:	G620111
Carrot fresh cooked	Value:	G620121
cassava leaves fresh cooked with groundnuts	Value:	Z000049
caterpillars (vinkhulubala)	Value:	3
Chicken	Value:	21
Chikanda	Value:	4
Chinese cabbage	Value:	20
Chinese cabbage fresh cooked	Value:	G321121
Chips (French-fried potatoes) (R)	Value:	_164700
Cola beverages (caffeinic)	Value:	N330000
Corn puffs	Value:	22
Cow's milk boiled	Value:	M110030
Dairy Products-Tinned or Fresh milk	Value:	5
Doughnuts (R)	Value:	_115001
Eggs Boiled	Value:	Z000031
Fish small dried with oil and tomatoes	Value:	Z000008
Fried sausage (R)	Value:	_025002
Groundnuts. Dried. raw Z	Value:	Z000015
Groundnuts. Powder T	Value:	Z000017
Impwa	Value:	18
Inswa	Value:	6
Kapenta (fish)	Value:	Z000018
Lumanda	Value:	7
Maize breakfast meal Z	Value:	Z000025

Maize Porridge with sugar	Value:	Z000046
Maize roller meal Z	Value:	Z000026
Maize samp	Value:	8
Mango fresh	Value:	F516111
Manioc (cassava)	Value:	K410000
Mice (mbeba)	Value:	9
Munkoyo	Value:	10
Mushrooms fresh cooked	Value:	K700121
Natural youghurt	Value:	11
Nshima Maize	Value:	Z000022
Nshima, maize and sorghum	Value:	Z000029
Nshima, sorgum and millet	Value:	Z000030
Okra Lady fingers	Value:	12
Onion	Value:	19
Onions fresh cooked	Value:	G480121
Orange fresh	Value:	F603111
Papaya fresh	Value:	F517111
Peanut butter	Value:	13
Pizza	Value:	_164100
Pumpkin leaves cooked with groundnuts	Value:	Z000063
Pumpkin leaves relish without oil	Value:	Z000006
Rape fresh boiled	Value:	Z000010
Rice parboiled cooked	Value:	C359022
Salt	Value:	14
Shake and Sip (maheu)	Value:	Z000040
Sour milk	Value:	15
Sugar T	Value:	Z000012
Sunflower oil	Value:	16

Sweet potato fresh EP	Value: Z000038
Sweet potato leaves boiled	Value: Z000003
Sweet potato leaves cooked with groundnuts	Value: Z000052
Tomato red fresh	Value: G561111
Tomatoes fresh cooked	Value: G560121
Water	Value: 17
Watermelon fresh	Value: F509111
Wholemeal bread	Value: B101000
Other (please specify!)	Value: 998

abc * If OTHER, please specify:

Please complete Sections: C, D and E ONLY after you have written down the all the food and drinks ${name} ate since waking up yesterday until ${name} went to sleep the next day.

* C. How was this food item prepared?

Raw / fresh	Value: 1
Boiled	Value: 2
Cooked	Value: 3
Roasted	Value: 4
Fried	Value: 5
Steamed	Value: 6
Baked	Value: 7
Smoked	Value: 8
Dried	Value: 9
Processed	Value: 10

Not applicable	Value: 999
No answer	Value: 997

1.0 * D. Amount / quantity of food/drink consumed:

* E. Unit of measurement:

g (grams)	Value: 1
ml (milliliters)	Value: 2

* E. Category:

Group 1A: Grains , roots, and tubers. i.e. maize meal porridge, maize meal and cassava porridge, sorghum and millet porridge, bread, rice, munkoyo and other foods made from grains.	Value: 1a
Group 1B: White potatoes, sweet potatoes, white yams, cassava, chikanda and other foods made from roots.	Value: 1b
Food item was not mentioned in or does not fit any of the food categories above.	Value: 10
Group 2: Legumes and nuts. i.e. beans, ground nuts, peas, lentils and seeds.	Value: 2
Group 3: Milk and milk products (e.g.: cheese, milk (fresh & processed), sour milk, yoghurt, etc.)	Value: 3a
Group 4: Flesh foods. i.e. organ meats (Liver, Kidney, Heart, Tongue) and any meat (Beef, Pork, Lamb, Goat, Chicken or Duck).	Value: 4
Group 5: Eggs	Value: 5
Group 6A: Vitamin A rich fruit and vegetables yellow/orange colour in the inside (e.g. papaya, pumpkin, carrots, squash, or orange-fleshed sweet potatoes, guava, mangoes)	Value: 6a
Group 6B: Vitamin A rich vegetables. i.e. dark green vegetables, bondwe, chibwabwa, kalembula, Chinese cabbage	Value: 6b
Group 6C: other vegetables e.g. impwa, cucumber, cabbage	Value: 6d
Group 7: Other fruit and vegetables. i.e. wild fruit (Mfungo, Mutungulu, Chisongole) or any other wild fruit or vegetables.	Value: 7
Group 8A: Others (i.e. any oil, fats, or butter or foods made with any of these)	Value: 8

Group 8B: Others (i.e. any sugary foods (Chocolates, Sweets, Candies, Pastries, Cakes or Biscuits))	Value: 8b
Group 8C: Others (i.e. condiments for flavor (Chillies, Spices, Herbs, or Fish powder))	Value: 8c
Group 9: Beverages (i.e. tea, coffee, etc.)	Value: 9

1+1 count(${questions_male})

Number of food items the male household member ate yesterday: ${C5_8}

Household Dietary Diversity

In the past one week, how many times were the following food types eaten by members of your household, either as part of meals or snacks?

* Fresh Fish (small)

Yes	Value: 1
No	Value: 0

* Frequency of consumption:

Everday, more than once a day	Value: 1
Everyday, once a day	Value: 2

Three to four days	Value: 3
One or two days only	Value: 4

* Fresh Fish (large)

Yes	Value: 1
No	Value: 0

* Frequency of consumption:

Everday, more than once a day	Value: 1
Everyday, once a day	Value: 2
Three to four days	Value: 3
One or two days only	Value: 4

* Dried Fish (small)

Yes	Value: 1
No	Value: 0

* Frequency of consumption:

Everday, more than once a day	Value: 1
Everyday, once a day	Value: 2
Three to four days	Value: 3
One or two days only	Value: 4

* Dried Fish (medium)

Yes	Value: 1
No	Value: 0

* Frequency of consumption:

Everday, more than once a day	Value: 1
Everyday, once a day	Value: 2
Three to four days	Value: 3
One or two days only	Value: 4

* Dried Fish (large)

Yes	Value: 1
No	Value: 0

* Frequency of consumption:

Everday, more than once a day	Value: 1
Everyday, once a day	Value: 2
Three to four days	Value: 3
One or two days only	Value: 4

* Smoked fish (small)

Yes	Value: 1
No	Value: 0

* Frequency of consumption:

Everday, more than once a day	Value: 1
Everyday, once a day	Value: 2
Three to four days	Value: 3
One or two days only	Value: 4

* Smoked fish (medium/large)

Yes	Value: 1
No	Value: 0

* Frequency of consumption:

Everday, more than once a day	Value: 1
Everyday, once a day	Value: 2
Three to four days	Value: 3
One or two days only	Value: 4

* Salted Fish

Yes	Value: 1
No	Value: 0

* Frequency of consumption:

Everday, more than once a day	Value: 1
Everyday, once a day	Value: 2
Three to four days	Value: 3
One or two days only	Value: 4

* Beef - steak meat, mixed cut

Yes	Value:	1
No	Value:	0

* Frequency of consumption:

Everday, more than once a day	Value:	1
Everyday, once a day	Value:	2
Three to four days	Value:	3
One or two days only	Value:	4

* Beef- organ meat, offals (Ichifu)

Yes	Value:	1
No	Value:	0

* Frequency of consumption:

Everday, more than once a day	Value:	1
Everyday, once a day	Value:	2
Three to four days	Value:	3
One or two days only	Value:	4

* Beef- other parts, e.g. head, hoof, etc.

Yes	Value:	1
No	Value:	0

* Frequency of consumption:

Everday, more than once a day	Value:	1
Everyday, once a day	Value:	2
Three to four days	Value:	3
One or two days only	Value:	4

* Insects (caterpillar, ants, inswa, Nshonkonono)

Yes	Value:	1
No	Value:	0

* Frequency of consumption:

Everday, more than once a day	Value:	1
Everyday, once a day	Value:	2

Three to four days	Value: 3
One or two days only	Value: 4

* Poultry

Yes	Value: 1
No	Value: 0

* Frequency of consumption:

Everday, more than once a day	Value: 1
Everyday, once a day	Value: 2
Three to four days	Value: 3
One or two days only	Value: 4

* Eggs

Yes	Value: 1
No	Value: 0

* Frequency of consumption:

Everday, more than once a day	Value: 1
Everyday, once a day	Value: 2
Three to four days	Value: 3
One or two days only	Value: 4

* Pulses and nuts (beans, cowpeas, groundnuts, pigeon peas)

Yes	Value: 1
No	Value: 0

* Frequency of consumption:

Everday, more than once a day	Value: 1
Everyday, once a day	Value: 2
Three to four days	Value: 3
One or two days only	Value: 4

* Fresh milk and other milk products. For example: Sour milk (mabisi atemile), Longlife milk (mabisi asikuwa, amwa mapepa amu zilili), Powdered milk (mabisi abupi), Yoghurt.

Yes	Value: 1
No	Value: 0

* Frequency of consumption:

Everday, more than once a day	Value: 1
Everyday, once a day	Value: 2
Three to four days	Value: 3
One or two days only	Value: 4

* Infant Formula (nan, etc.)

Yes	Value: 1
No	Value: 0

* Frequency of consumption:

Everday, more than once a day	Value: 1
Everyday, once a day	Value: 2
Three to four days	Value: 3
One or two days only	Value: 4

* Cereal (nshima, rice, green maize)

Yes	Value: 1
No	Value: 0

* Frequency of consumption:

Everday, more than once a day	Value: 1
Everyday, once a day	Value: 2
Three to four days	Value: 3
One or two days only	Value: 4

* Roots and Tubers. (cassava, sweet potatoes, Irish potatoes)

Yes	Value: 1
No	Value: 0

* Frequency of consumption:

Everday, more than once a day	Value:	1
Everyday, once a day	Value:	2
Three to four days	Value:	3
One or two days only	Value:	4

* Wheat products (bread, fritters, doughnuts, cakes)

Yes	Value:	1
No	Value:	0

* Frequency of consumption:

Everday, more than once a day	Value:	1
Everyday, once a day	Value:	2
Three to four days	Value:	3
One or two days only	Value:	4

* Vegetables (rape, chiwawa, kalembula, spinach)

Yes	Value:	1
No	Value:	0

* Frequency of consumption:

Everday, more than once a day	Value: 1
Everyday, once a day	Value: 2
Three to four days	Value: 3
One or two days only	Value: 4

* Vegetables (fisashi, impwa)

Yes	Value: 1
No	Value: 0

* Frequency of consumption:

Everday, more than once a day	Value: 1
Everyday, once a day	Value: 2
Three to four days	Value: 3
One or two days only	Value: 4

* Vegetables (cabbage)

Yes	Value: 1
No	Value: 0

* Frequency of consumption:

Everday, more than once a day	Value: 1
Everyday, once a day	Value: 2
Three to four days	Value: 3
One or two days only	Value: 4

* Vegetables (carrots, butternut, pumpkin, beetroot)

Yes	Value: 1
No	Value: 0

* Frequency of consumption:

Everday, more than once a day	Value: 1
Everyday, once a day	Value: 2
Three to four days	Value: 3
One or two days only	Value: 4

* Fresh fruit (guavas, mangoes, orange, tangerines)

Yes	Value: 1
No	Value: 0

* Frequency of consumption:

Everday, more than once a day	Value: 1
Everyday, once a day	Value: 2
Three to four days	Value: 3
One or two days only	Value: 4

* Beverages (coffee, tea, cocoa)

Yes	Value: 1
No	Value: 0

* Frequency of consumption:

Everday, more than once a day	Value: 1
Everyday, once a day	Value: 2
Three to four days	Value: 3
One or two days only	Value: 4

* Sugar, Jam, Marmalade, Honey

Yes	Value: 1
No	Value: 0

* Frequency of consumption:

Everday, more than once a day	Value: 1
Everyday, once a day	Value: 2
Three to four days	Value: 3
One or two days only	Value: 4

* Oils and Fats (butter, margerine, and othe edible fats and oils)

Yes	Value: 1
No	Value: 0

* Frequency of consumption:

Everday, more than once a day	Value: 1
Everyday, once a day	Value: 2
Three to four days	Value: 3
One or two days only	Value: 4

* What is the main source of food consumed in this household?

Food acquired from purchases	Value: 1
Food caught / produced by the household	Value: 2
Gifts	Value: 3
Food assistance	Value: 4
Exchanged for other item (barter)(please specify)	Value: 5
Other (please specify!)	Value: 998

abc * If Other, please specify:

Section D: Household Fish consumption

* Has the household eaten fish during the last 24 hours?

Yes	Value: 1
No	Value: 0

* Tick the fish species that was consumed by any member of the household yesterday. If more, please tick the + button and repeat!

Nchenga	Value: 1
Mintesa / Nyanse	Value: 2
Milamba / Milonge (Bubble Fish)	Value: 3
Buka Buka Fish	Value: 4
Kapenta	Value: 5
Chisense	Value: 6
Mpulungu Chisense	Value: 7
Siavonga Chisense	Value: 8
Kababa (small fish)	Value: 9
Nkolonkolo	Value: 10
Dagga	Value: 11
Bream	Value: 12
Other (please specify!)	Value: 998

abc * Please specify!

* Do you know which bream species?

Yes	Value: 1
No	Value: 0

* Select species:

Three-spotted Bream	Value: 1
Nile Bream	Value: 2
Green-headed Bream	Value: 3
Redbreast Bream	Value: 4

* How did you acquire the fish?

Fish acquired from purchases	Value: 1
Fish caught or produced by a household member	Value: 2
Gift	Value: 3
Food assistance	Value: 4
Exchanged for other item (barter)(please specify)	Value: 5
Other (please specify!)	Value: 998

abc * Please specify the item of barter

abc * Please specify

* In what form was it acquired?

Fresh	Value: 1
Dried	Value: 2
Fried	Value: 3
Salted and dried	Value: 4
Smoked	Value: 5
Roasted	Value: 6

* Wich portion was acquired?

Whole fish	Value: 1
Chunk of fish	Value: 2
Small fish (whole)	Value: 3

* Which way of preparation?

Raw	Value: 1
Fried	Value: 2
Boiled / cooked	Value: 3
Steamed	Value: 4
Dried	Value: 5

Roasted	Value: 6
Fish was pre-prepared	Value: 7

Fish consumption patterns (1)

* Do all household members consume all types of fish species?

Yes	Value: 1
No	Value: 0

* Tick the household member / group who does / do not consume fish. If more groups, please tick the + button and repeat for every single group!

Children	Value: 1
Pregnant women	Value: 2
Elderly women	Value: 3
Lactating mothers	Value: 4
Other (please specify!)	Value: 998

abc * If other, please specify!

* Which fish species do they not consume?

Nchenga	Value:	1
Mintesa / Nyanse	Value:	2
Milamba / Milonge (Bubble Fish)	Value:	3
Buka Buka Fish	Value:	4
Kapenta	Value:	5
Chisense	Value:	6
Mpulungu Chisense	Value:	7
Siavonga Chisense	Value:	8
Kababa (small fish)	Value:	9
Nkolonkolo	Value:	10
Dagga	Value:	11
Bream	Value:	12
Other (please specify!)	Value:	998

abc * If other, please specify!

* Which part of the fish do they not consume?

Head	Value:	1
Tail	Value:	2
Filet	Value:	3
Edible offals	Value:	4
Not applicable	Value:	999
No answer	Value:	997

Fish consumption patterns (2)

* In your community, are pregnant and lactating women allowed to consume all types of fish species?

Yes	Value: 1
No	Value: 0

* If no, what are the reasons that hinder the consumption of fish by these groups of women?

Gives rashes	Value: 1
Connected to evil spirits	Value: 2
Use of unhealthy preservatives by traders	Value: 3
Fish has unpleasant smell	Value: 4
Children can get injured by the bones	Value: 5
Considered as food for low class people	Value: 6
Other (please specify!)	Value: 998

abc * If other, please specify!

Preferred fish

What are the top 3 mostly consumed fish species?

* Select rank 1:

Nchenga	Value: 1
Mintesa / Nyanse	Value: 2
Milamba / Milonge (Bubble Fish)	Value: 3
Buka Buka Fish	Value: 4
Kapenta	Value: 5
Chisense	Value: 6
Mpulungu Chisense	Value: 7
Siavonga Chisense	Value: 8
Kababa (small fish)	Value: 9
Nkolonkolo	Value: 10
Dagga	Value: 11
Bream	Value: 12
Other (please specify!)	Value: 998

abc

* If other, please specify!

* Why do you consume / prefer this fish?

Price (cheaper than other fish)	Value: 1
Taste (tastes better than other fish)	Value: 2
Availability (easily available)	Value: 3
Comfort (easy to prepare and cook)	Value: 4
Other (please specify!)	Value: 998

abc * If other, please specify!

* Select rank 2:

Nchenga	Value: 1
Mintesa / Nyanse	Value: 2
Milamba / Milonge (Bubble Fish)	Value: 3
Buka Buka Fish	Value: 4
Kapenta	Value: 5
Chisense	Value: 6
Mpulungu Chisense	Value: 7
Siavonga Chisense	Value: 8
Kababa (small fish)	Value: 9
Nkolonkolo	Value: 10
Dagga	Value: 11
Bream	Value: 12
Other (please specify!)	Value: 998

abc * If other, please specify!

* Why do you consume / prefer this fish?

Price (cheaper than other fish)	Value: 1

Taste (tastes better than other fish)	Value: 2
Availability (easily available)	Value: 3
Comfort (easy to prepare and cook)	Value: 4
Other (please specify!)	Value: 998

abc * If other, please specify!

* Select rank 3:

Nchenga	Value: 1
Mintesa / Nyanse	Value: 2
Milamba / Milonge (Bubble Fish)	Value: 3
Buka Buka Fish	Value: 4
Kapenta	Value: 5
Chisense	Value: 6
Mpulungu Chisense	Value: 7
Siavonga Chisense	Value: 8
Kababa (small fish)	Value: 9
Nkolonkolo	Value: 10
Dagga	Value: 11
Bream	Value: 12
Other (please specify!)	Value: 998

abc * If other, please specify!

* Why do you consume / prefer this fish?

Price (cheaper than other fish)	Value:	1
Taste (tastes better than other fish)	Value:	2
Availability (easily available)	Value:	3
Comfort (easy to prepare and cook)	Value:	4
Other (please specify!)	Value:	998

abc * If other, please specify!

Section E: Anthropometric Measurements

Anthropometry-Children

Measure all children younger than 5 years of age. If the mother of the child is not a household member (for example, mother is deceased) put 55. In case the household has more than one child, please repeat this for every eligible child under 5 years of age by pressing the + button to open up a new form. Repeat this as needed!

abc * Name of child:

abc * Name of mother:

* Child's date of birth:

* Date of birth confirmed with birth certificate or health card?

Yes	Value:	1
No	Value:	0

1.0 * Weight in kg:

1.0 * Height in cm:

* How was this child's height measured?

Lying down	Value:	1
Standing up	Value:	2
No measurement was taken	Value:	3

abc * Reason for not being measured?

* Is this child still breast fed?

Yes	Value:	1
No	Value:	0

Anthropometrics of the mother or the permant care taker of the child

Measure mother 14-49 years of age! In case the household has more than one care giver / mother, please repeat this for every eligible mother by pressing the + button to open up a new form. Repeat this as needed!

abc * Name:

* Are you pregnant?

Yes	Value:	1
No	Value:	0

* Are you lactating?

Yes	Value:	1
No	Value:	0

1.0 * Height in cm

1.0 * Waist circumference in cm

1.0 * Weight in kg

1+1 ${E14} div ((${E12} div 100)*(${E12} div 100))

BMI of ${E_name_women}: ${E15}

Section F: Health

Please fill the following forms for the child and / or the infant. Make sure that information is collected of those children that the recall was done for earlier on.

Health of the Child (2-5 years of age)

* Has ${name_child} suffered from any illnesses in the last two weeks?

Option	Value
Yes	1
No	0

* From which illness has ${name_child} suffered from in the last two weeks?

Option	Value
Diarrhea	1
Fever	2
Malaria	3
Cough and flu	4
Other (please specify!)	998

abc * If other, please specify!

Health of the Infant (< 23 months of age)

* Has ${name_infant} suffered from any illnesses in the last two weeks?

Yes	Value: 1
No	Value: 0

* From which illness has ${name_infant} suffered from in the last two weeks?

Diarrhea	Value: 1
Fever	Value: 2
Malaria	Value: 3
Cough and flu	Value: 4
Other (please specify!)	Value: 998

abc * If other, please specify!

Section G: Final remarks

* Any comments or special remarks to be added?

Yes	Value:	1
No	Value:	0

abc

* Please, list them here:
